# Advanced-stage breast cancer diagnosis and its determinants in Ethiopia: a systematic review and meta-analysis

**DOI:** 10.1186/s12905-024-03133-9

**Published:** 2024-05-11

**Authors:** Amare Zewdie, Tadele Derbew Kassie, Tadele Fentabel Anagaw, Elyas Melaku Mazengia, Sintayehu Shiferaw Gelaw, Eneyew Talie Fenta, Habitu Birhan Eshetu, Natnael Kebede, Eyob Ketema Bogale

**Affiliations:** 1 Department of Public Health, College of Medicine and Health Science, Wolkite University, Wolkite, Ethiopia; 2 Department of Public Health, College of Medicine and Health Science, Debre Markos University, Debre Markos, Ethiopia; 3 Health Promotion and Behavioural science department, College of medicine and health science, Bahir Dar University, Bahir Dar, Ethiopia; 4 Department of Health Promotion and Health Behaviour, Institute of Public Health, College of Medicine and Health Sciences, University of Gondar, PO.Box.196, Gondar, Ethiopia; 5 Department of Public Health, College of Medicine and Health Sciences, Injibara University, Injibara, Ethiopia; 6 Department of Health Promotion, School of Public Health, College of Medicine and Health Sciences, Wollo University, Dessie, Ethiopia

**Keywords:** Breast cancer, Delayed diagnosis, Advanced-stage cancer, Cancer diagnosis, Ethiopia

## Abstract

**Introduction:**

Worldwide, breast cancer is the primary cause of illness and death. Unless early detected and treated breast cancer is a life-threatening tumor. Advanced-stage presentation is greatly linked with short survival time and increased mortality rates. In Ethiopia nationally summarized evidence on the level of advanced-stage breast cancer diagnosis is scarce. Therefore, this systematic review and meta-analysis aimed to determine the pooled prevalence of advanced-stage breast cancer diagnosis and its determinants in Ethiopia.

**Method:**

By following PRISMA guidelines, a systematic review and meta-analysis were carried out. To include relevant publications, a broad literature search was conducted in the African Online Journal, PubMed, Google Scholar, and Embase which are published until last search date; June 15, 2023. To prevent further duplication this review was registered in PROSPERO database with ID no of CRD42023435096. To determine the pooled prevalence, a weighted inverse variance random effect model was applied. I^2^ statistics and the Cochrane Q-test were computed to determine heterogeneity. To evaluate publication bias, a funnel plot, and Egger’s regression test were used.

**Result:**

A total of 924 articles were sought and finally 20 articles were included in this review. The pooled prevalence of advanced-stage breast cancer diagnosis in Ethiopia was 72.56% (95%CI; 68.46-76.65%). Use of traditional medicine as first choice (AOR = 1.32, 95% CI: (1.13–1.55)), delay of > 3 months in seeking care (AOR = 1.24, 95% CI: (1.09–1.41)), diagnosis or health system delay of > 2 months (AOR = 1.27, 95% CI: (1.11–1.46)), rural residence (AOR = 2.04, 95% CI: (1.42 − 2.92)), and chief complaint of a painless breast lump (AOR = 2.67, 95% CI: (1.76–4.06)) were significantly associated to advanced-stage diagnosis.

**Conclusion:**

In Ethiopia, more than two-thirds of breast cancer cases are diagnosed at an advanced stage. Use of traditional medicine before diagnostic confirmation, delay in seeking care, health system delay, rural residence, and chief complaint of painless breast lump were positively associated with an advanced-stage diagnosis. Policymakers and program designers give great focus to those delays so as to seek and access modern diagnosis and treatment as early as possible specifically focusing on those who are rurally residing.

**Supplementary Information:**

The online version contains supplementary material available at 10.1186/s12905-024-03133-9.

## Introduction

Globally, breast cancer accounts for the majority of cancer-related morbidity and death with 2.3 million new cases and 685,000 deaths resulting from it in 2020 [1]. In a significant portion of the global population, an increasing trend in the burden of breast cancer was observed [2]. Despite of poor diagnosis system in Africa breast cancer is responsible for one in four diagnosed cancers and one in five cancer deaths in women [3]. In Ethiopia breast cancer is the most frequently diagnosed cancer and the leading cause of cancer death in women, with an estimated 15,244 newly diagnosed cases and 8159 deaths in 2018 [4]. The most commonly identified risk factors for the occurrence of breast cancer in Ethiopia were family history of breast cancer, early menarche, being in post-menopause, and not ever breast feed [5].

Unless early detected and treated breast cancer is a life-threatening cancer that results in both local and distant metastases and end up with death [6]. Evidence showed that delayed breast cancer diagnosis which might result from both patient delay to seek care and health system delay (delay within the health care system) is associated with more advanced-stage cancers at diagnosis, thus resulting in poorer chances for survival [7]. Advanced or late-stage presentation (stages III and IV) is greatly linked with short survival time and increased mortality rates [8]. Patients diagnosed at the advanced stage of breast cancer receive palliative care and have poorer prognoses than those diagnosed at the early stage [9]. Evidence showed that the five-year survival for patients diagnosed at stage IV was nearly threefold lower than that of patients diagnosed at stage I [10].

Breast cancer’s mortality rate is declining in several developed countries as a result of early diagnosis and better-quality treatment. In contrast, the death rate is increasing in developing countries as a consequence of increasing risk factors for developing the disease and a poor system of early diagnosis and treatment [11]. In developed countries, more than 70% of breast cancer patients are diagnosed at an early stage (stages I and II), and the prognosis is good and mortality is lower; however, in developing countries like Africa, where the infrastructure for early detection is very poor, breast cancer is frequently diagnosed at late stages with only 20–50% of patients diagnosed in early stages of the disease which makes breast cancer the most deadly cancer in the continent [12, 13]. Ethiopia is a member of the continent, and a large proportion of breast cancer patients face longer time delays in diagnosis and treatment [14, 15].

Several factors were also identified as barriers to early diagnosis and treatment of breast cancer in Ethiopia. Belief in traditional medicine and religious practices for treatment, Lack of breast self-examination and lack of social and financial support, misdiagnosis of breast cancer, long distance to referral facilities, long waiting times for diagnostic tests, and high cost of diagnostic services were the most frequently raised patient and health-system related barriers [14–16].

Until now, many studies in Ethiopia have discovered a highly varying level of advanced-stage breast cancer diagnoses in various regions of the nation. As far as we know, in Ethiopia there were no systematic reviews or meta-analyses that can provide summarized evidence on the level of advanced-stage breast cancer diagnosis and its determinants; despite it was burning issue and public health problem which makes it more important to explore the topic and take appropriate action. Therefore, the current meta-analysis intended to determine the overall prevalence of advanced-stage breast cancer diagnosis and identify its contributing factors in Ethiopia.

This systematic review and meta-analysis’s findings provide evidence that can be utilized to develop and carry out actions to lower the level of advanced-stage breast cancer diagnosis in the country. The review finds the contributing factors to advanced-stage breast cancer diagnosis, it enables respective stakeholders to target and design evidence-based interventions. Since there hasn’t been a review and meta-analysis of the literature on this subject area, this study can also be used as a baseline comparison. Moreover, it could spark fresh ideas for future research on the topic.

## Method

### Study design

On the prevalence of advanced-stage breast cancer diagnosis and its determinants in Ethiopia, a systematic review and meta-analysis were carried out. The standards for Preferred Reporting Items for Systematic Review and Meta-Analysis (supplementary Table 1) were adhered to. Checklists that provide guidance for conducting and reporting systematic reviews and meta-analyses are part of the PRISMA procedure. This method improves the accuracy and transparency of reviews across a range of fields, including medicine [17, 18].

### Study setting

This systematic review and meta-analysis incorporate studies on advanced stage breast cancer diagnosis in Ethiopia. One of the low-income countries in the Horn of Africa, Ethiopia is projected to have 123.4 million residents in 2022, 133.5 million in 2032, and 171.8 million in 2050 [19].

## Search strategies and sources of information

To prevent further duplication, we have looked up published or current projects relating to the topic in the PROSPERO database (http://www.library.ucsf.edu). The results showed that there were neither continuing nor published articles on this topic. Subsequently, the PROSPERO database hosted this systematic review and meta-analysis with ID CRD42023435096. We used the international databases PubMed, Embase, Google Scholar, and African Online Journal to search a comprehensive body of literature and retrieve related articles. Using online databases, search phrases were created in accordance with PICO requirements. Keywords and Medical Subject Headings (MeSH) were generated using the Boolean operators “AND” and “OR“(supplementary file 3). Grey literature was also searched using Google by exploring the research repository of several universities in the country through their online address using the study topic as a search term.

### Eligibility criteria

Studies on advanced-stage breast cancer diagnosis and its determinants in Ethiopia that are written in English are eligible to be included in this systematic review and meta-analysis; there are no restrictions on race, gender, or publication date (until the last search date June 15, 2023). Articles without full abstracts or texts, as well as those reported outside of the outcome of interest, were excluded. Newspaper articles, reviews, meta-analyses, editorials, and other reporting from popular media were excluded at each screening step.

### Outcome measurements

From the two outcomes of this study; the first is the prevalence of advanced-stage breast cancer diagnoses. Its definition is the percentage of individuals who first presented with advanced stages of breast cancer (stages III and IV). As a result, the included studies evaluated the disease status of the study participants and classified them as either having an advanced or early stage of breast cancer. Following analysis, the response was given as the prevalence of advanced-stage breast cancer diagnoses. Determinants of advanced-stage breast cancer diagnosis were the secondary outcome.

### Data extraction

In order to eliminate duplicate studies, every study retrieved from the databases under consideration was exported to Endnote version X8. Subsequently, every study was exported into an Excel spreadsheet. Using a standardized data extraction form that was adapted from the Joanna Briggs Institute (JBI) data extraction format. Four authors (TDK, TFA, EMM and SSG) assessed the quality of each study (i.e. methodological quality, sample selection, sample size, and statistical analysis of the study). In the case of disagreement between three authors; another four authors (NK, HBE, ETF and EKB) involved and resolved the disagreement. The first author (AZ) facilitate the overall extraction and quality assessment. The following was included in the data extraction format for the first outcome (prevalence): primary author, year of publication, regions, study area, sample size, and prevalence with a 95% confidence interval. Using a 2 by 2 table structure, we extracted data for the second outcome (associated factors to advanced-stage diagnosis).

### Quality assessment

The Newcastle Ottawa Quality Assessment Scale for cross-sectional studies was used to rate the quality of each included study [20] (Supplementary Table 2). The methodological quality, sample selection, sample size, result comparability, and statistical analysis of each study were evaluated by two authors (AZ, EMM). When two authors disagreed, two more authors (HBE, NK) got involved, conversed about it, and worked out a solution.

### Data processing and analysis

For analysis, the data in Microsoft Excel spreadsheet format was imported into STATA version 17. The pooled prevalence of delayed breast cancer presentation in Ethiopia was then estimated using a weighted inverse variance random effect model. The pooled prevalence of late-stage breast cancer presentation with a 95% confidence interval was displayed using a forest plot approach. The log odds ratio for each factor was determined in order to find the determinants of an advanced-stage breast cancer diagnosis. This effect measure was then utilized to construct the pooled AOR.

### Heterogeneity test and publication bias

The I^2^ statistics and the Cochrane Q-test were used to determine the degree of heterogeneity across all studies. As a result, mild heterogeneity is defined as I^2^ results between 0% and 40%, moderate heterogeneity as between 40% and 70%, and significant heterogeneity as between 70% and 100% [21]. To evaluate publication bias, the Eggers test and funnel plot were employed. There was no publication bias when the p-value was greater than 0.05.

## Result

**Fig. 1:**
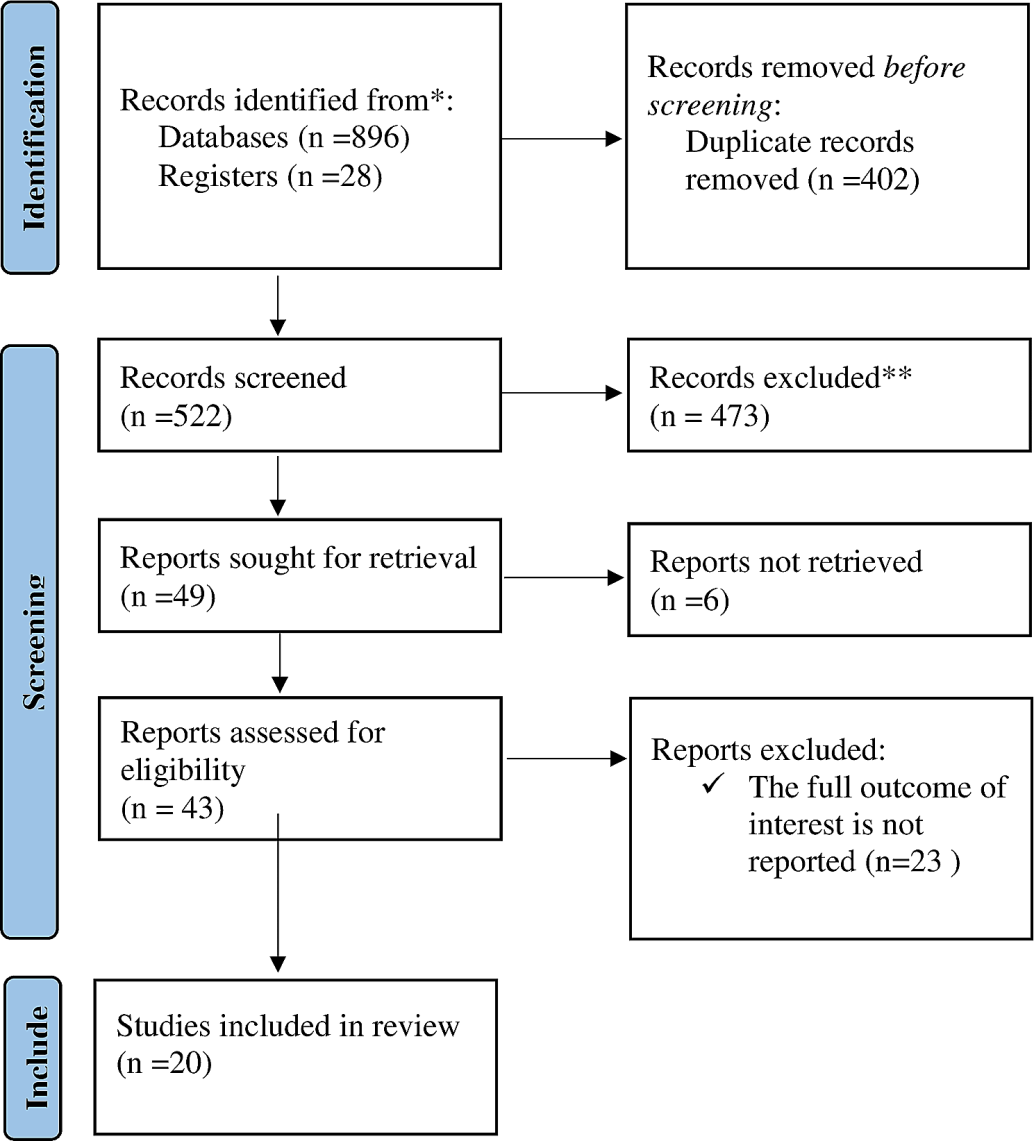
Flow chart of study selection for systematic review and meta-analysis on advanced-stage breast cancer diagnosis and its determinant in Ethiopia, 2023

Using our search method, 924 articles were found in the databases; PubMed, Embase, Google Scholar, and African Journals. There were 402 duplicate articles excluded. Following evaluation, from the remaining 522 articles; (*n* = 398) articles were excluded based on their titles, and (*n* = 81) articles were excluded based on their abstracts. Then 23 additional full-text papers were excluded for the aforementioned reason after 43 full-text articles had been retrieved and evaluated for inclusion criteria. As a result, the final systematic review and meta-analysis are performed on 20 studies that meet the inclusion criteria. (Fig. 1).

Those included studies comprised a total of 5,333 participants and found a 50.5–91.9% prevalence of advanced-stage breast cancer diagnosis. The Newcastle Ottawa Quality Assessment scale score of the included research ranges from 7 to 9, which was good (Table 1).

**Table 1:** Characteristics of included studies in the systematic review and meta-analysis on advanced-stage breast cancer diagnosis and its determinants in Ethiopia

S.no	Author	Period	Region	Study design	Sample	Age	Prevalence (%)	Study quality
1	Gebremariam, et al. [22].	2017–2018	Addis Ababa	Crossect	406	44.4	64.3	Good
2	Tesfaw, et al. [23]	2013–2017	SNNPR	Crossect	426	42.8	72.5	Good
3	Tesfaw, et al. [24]	2019–2020	Amhara	Crossect	371	40^*^	71.2	Good
4	Yoseph, et al. [25]	2019	SNNPR	Crossect	255	42^*^	86.3	Good
5	Abebe, et al. [26]	2018	Addis Ababa	Crossect	86	43.2	91.9	Good
6	Areri, et al. [27]	2012–2014	Addis Ababa	Crossect	627	42.61	69.9	Good
7	Assefa S [28].	2018–2021	Amhara	Crossect	132	38^*^	77.5	Good
8	Ayele, et al. [29]	2021	Addis Ababa	Crossect	205	43	80.5	Good
9	Belachew, et al. [30]	2015–2019	Oromia	Crossect	262	42.27	64.9	Good
10	Dagne, et al. [31]	2011–2012	Addis Ababa	Crossect	303	42.1	68	Good
11	Gebretsadik, et al. [32]	2013–2019	SNNPR	Crossect	475	38^*^	78.3	Good
12	Gemta, et al. [33]	2013–2015	Addis Ababa	Crossect	197	44.77	69.5	Good
13	Hassen, et al. [34]	2020	Amhara	Crossect	204	44.1	66.2	Good
14	Legese, et al. [35]	2017	Addis Ababa	Crossect	375	40^*^	64.8	Good
15	Muhammed, et al. [36]	2021	SNNPR	Crossect	150	37.4	66	Good
16	Shita, et al. [37]	2013–2018	SNNPR	Crossect	302	39^*^	83.4	Good
17	Solomon, et al. [38]	2010–2014	Addis Ababa	Crossect	136	40.6	59.6	Good
18	Tesfaw, et al. [39]	2016–2019	Amhara	Crossect	128	45^*^	85.2	Good
19	Teshome, et al. [40]	2018	Addis Ababa	Crossect	188	45^*^	50.5	Good
20	YOSEPH R [41].	2010–2014	Oromia	Crossect	108	45*	77.8	Good

Where * indicate median and the remaining are mean

### The magnitude of advanced-stage breast cancer diagnosis in Ethiopia

**Fig. 2:**
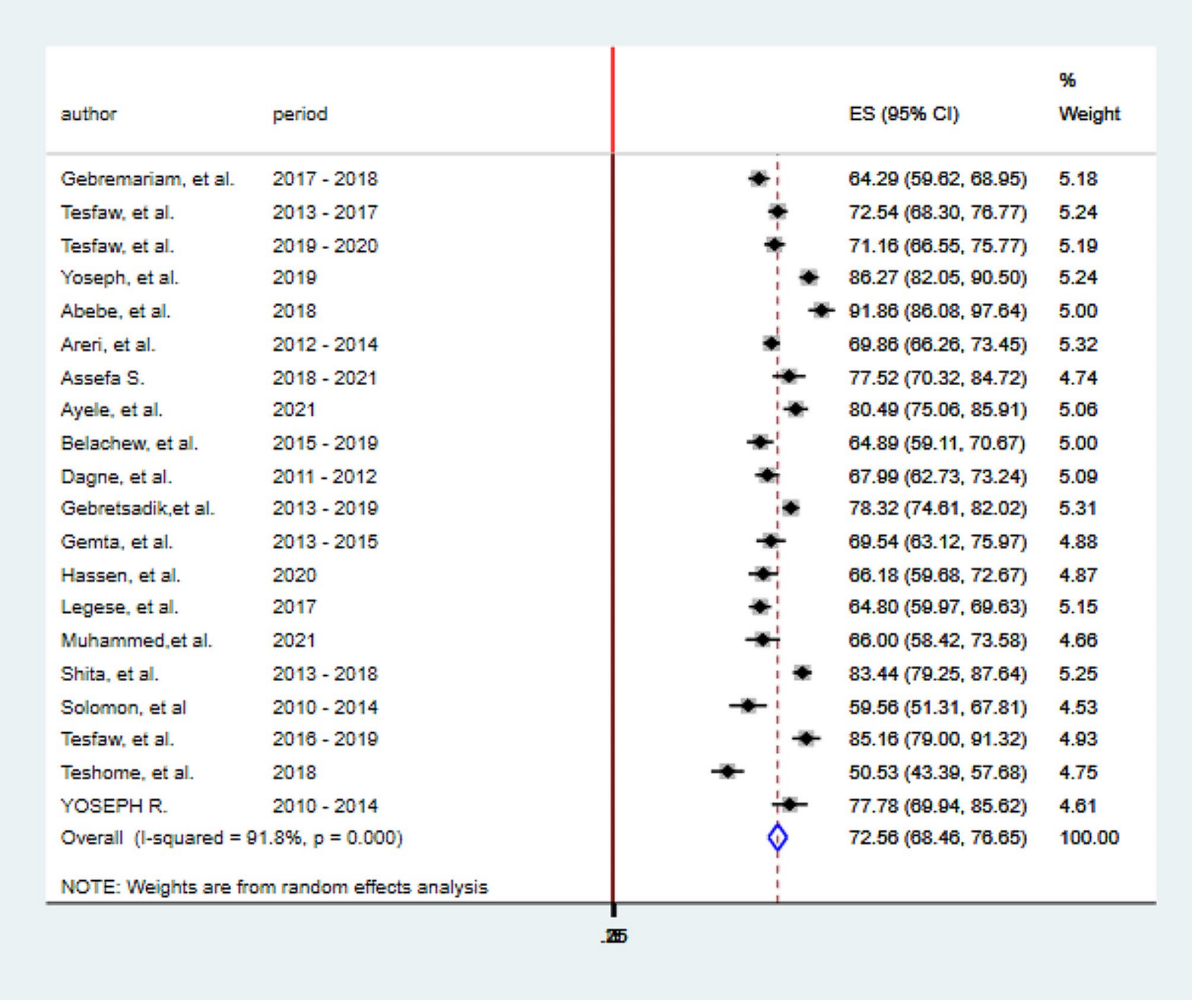
Forest plot showing the Pooled prevalence of advanced-stage breast cancer diagnosis in Ethiopia, 2023

The pooled prevalence of advanced-stage breast cancer diagnosis in Ethiopia was 72.56% (95%CI; 68.46-76.65%). The Cochrane heterogeneity index (I^2^ = 91.8%), *P* = 0.000, indicated significant heterogeneity of included studies (I2 > 70%). The results were displayed using a forest plot (Fig. 2).

### Publication bias

**Fig. 3:**
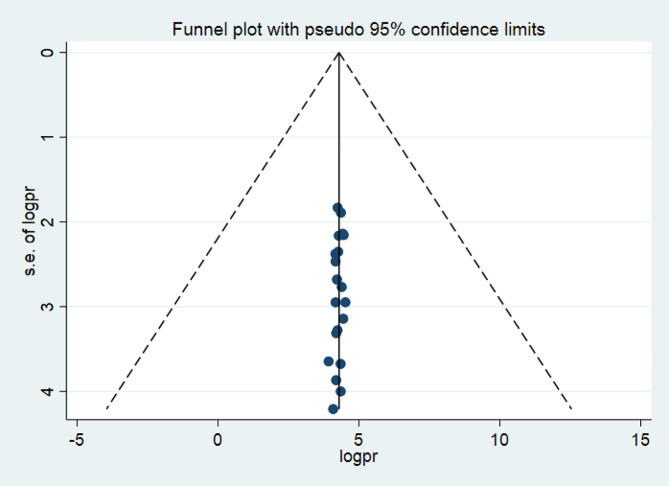
Funnel plot showing the symmetric distribution of articles on advanced-stage breast cancer diagnosis and its determinant in Ethiopia, 2023

A funnel plot was used to test for publication bias at a significance level lower than 0.05. No evidence of publication bias was confirmed by Egger’s regression test, since it was not statistically significant *P* = 0.221 (*p* > 0.05), as seen by the funnel plot (Fig. 3).

### Sensitivity analysis

**Fig. 4:**
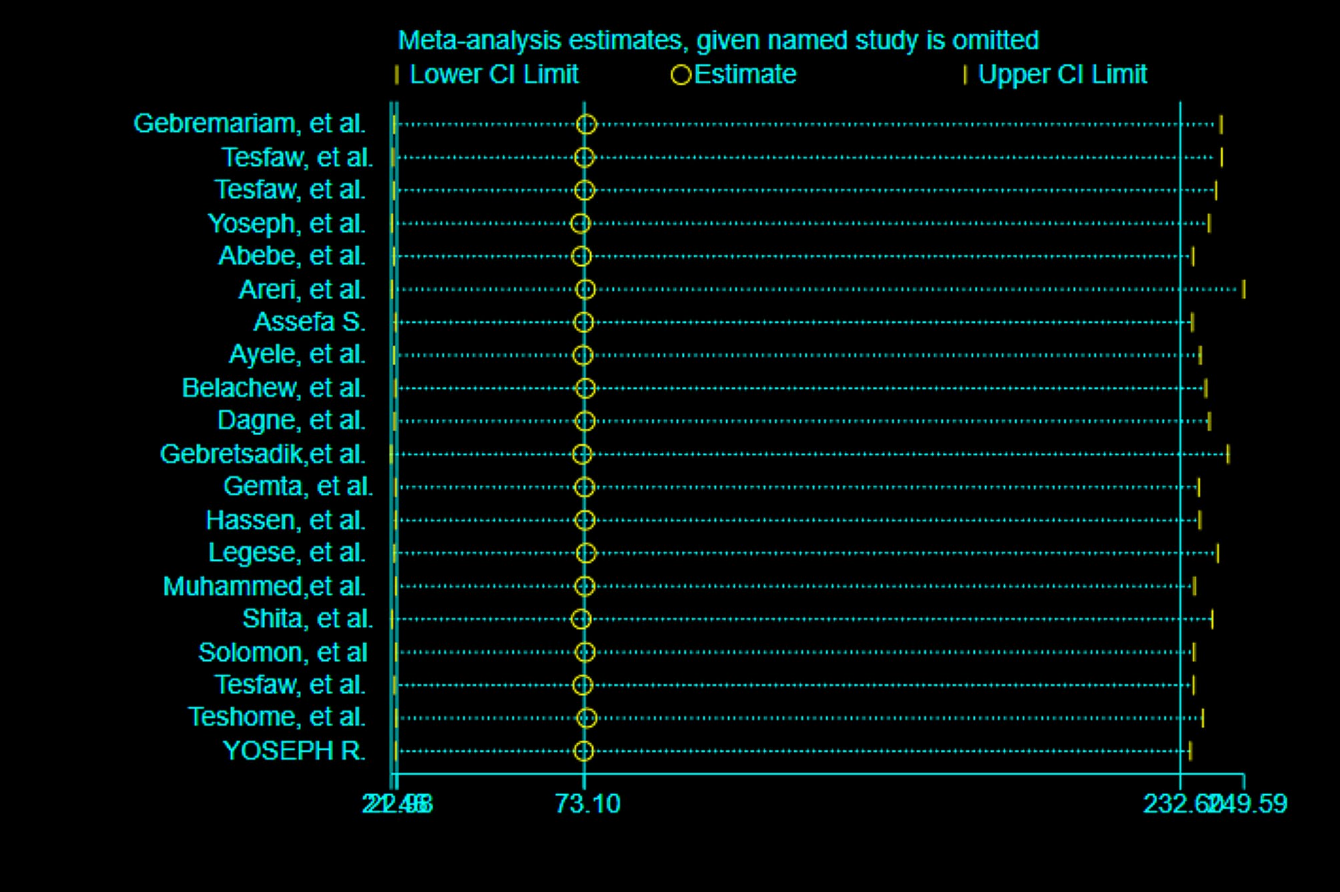
Sensitivity analysis of advanced-stage breast cancer diagnosis in Ethiopia, 2023

In this meta-analysis, no single study dominated the overall prevalence of advanced-stage breast cancer diagnosis, according to the results of a random-effects model (Fig. 4).

### Determinants of advanced-stage breast cancer diagnosis in Ethiopia

The identification of the determinants of advanced-stage breast cancer diagnosis in this systematic review and meta-analysis includes five factors that have been linked to the diagnosis of advanced-stage breast cancer in two or more primary studies. Consequently, first choice of traditional medicine, delay of > 3 months in seeking care, diagnosis or health system delay of > 2 months, rural residence, and chief complaint of painless breast lump were significantly associated with advanced-stage breast cancer diagnosis in Ethiopia. Patients who used traditional medicine before diagnostic confirmation were 1.32 times more likely to present with advanced-stage breast cancer as compared to their counterparts (AOR = 1.32, 95% CI: (1.13–1.55)). Similarly, Patients who had a delay of > 3 months in seeking care were 1.24 times more likely to be diagnosed at an advanced stage as compared to patients who sought care within 3 months of feeling symptoms (AOR = 1.24, 95% CI: (1.09–1.41)). Moreover, patients who faced a > 2-month diagnosis or health system delay were 1.27 times more likely to be present at the advanced stage as compared to patients who have not faced a health system delay (AOR = 1.27, 95% CI: (1.11–1.46)). Regarding residence, patients who are rurally residing were twice diagnosed at advanced-stage as compared to urban breast cancer patients (AOR = 2.04, 95% CI: (1.42 − 2.92)). Additionally, breast cancer patients who present with a chief complaint of painless breast lump were 2.67 times more likely to be diagnosed at an advanced stage as compared to patients who present with other chief complaints (AOR = 2.67, 95% CI: (1.76–4.06)) (Table 2).

**Table 2:** Factors associated with advanced-stage breast cancer diagnosis in Ethiopia

Variable	Authors	AOR	95%CI	Pooled AOR	95%CI of pooled AOR
Traditional medicine first choice	Gebremariam, et al. [22]	1.29	1.1–1.52	1.32	1.13–1.55
	Yoseph, et al. [25]	3.3	1.2–8.8		
Patient delay of > 3 months in seeking care	Gebremariam, et al. [22]	1.16	1.01– 1.34	1.24	1.09–1.41
	Tesfaw, et al. [23]	2.5	1.51–4.16		
	Tesfaw, et al. [24]	1.40	1.02–2.37		
Diagnosis or health system delay of > 2 months	Gebremariam, et al. [22]	1.24	1.07–1.43	1.27	1.11–1.46
	Tesfaw, et al. [23]	1.62	1.02–2.59		
Rural residence	Tesfaw, et al. [23]	2.37	1.45–3.86	2.04	1.42 − 2.92
	Tesfaw, et al. [24]	1.70	1.02–2.96		
Chief complaint of painless breast lump	Tesfaw, et al. [23]	3.01	1.49–6.07	2.67	1.76 – 4.06
	Tesfaw, et al. [24]	2.50	1.45–4.13		

## Discussion

Despite the incidence of breast cancer being lower in Africa, the mortality rate from this disease is higher than that of developed nations [42]. These uncorrelated statistics and facts imply the poor diagnostic and treatment setup in developing countries exposes victims of cancer to advanced-stage diagnosis and shorter survival. A recent systematic review that analyzed the level of delay in Africa and sub-Saharan Africa shows that a high proportion of breast cancer patients faced long delays and are diagnosed with late-stage disease [43, 44].

In Ethiopia, several primary studies also found a variable level of advanced-stage breast cancer diagnosis in the country, however, summarized evidence on the issue is lacking. Therefore, this systematic review and meta-analysis determined the magnitude of advanced-stage breast cancer diagnosis and its determinants among Ethiopian breast cancer patients. Accordingly, the pooled prevalence of advanced-stage breast cancer diagnosis in Ethiopia was 72.56% (95%CI; 68.46-76.65%). The finding was in line with studies done in 12 sub-Saharan countries [45], Libya [46], Nigeria [47], and northern Tanzania [48] in which more than two-thirds of breast cancer patients were diagnosed at an advanced stage (stage III and IV). This finding was significantly higher than two studies done in Iran [49, 50] 36.2% and 45.8%, northern Pakistan [51] 39%, Mexico [52] 47% and USA [53] 26.6% of breast cancer cases were diagnosed at an advanced stage. The possible discrepancy may be the difference in the health care setup and literacy level of the population in which the mentioned countries have better health care system that has advanced diagnostic facilities that can early diagnose and treat the disease. This large proportion of advanced-stage breast cancer diagnoses in Ethiopia implies strong effort at each level of the health system should be made to improve the early detection of breast cancer so as to diagnose and treat the disease early to improve the quality of life of the victims and decrease the disease’s related mortality in the country.

Decreasing the late-stage presentation of breast cancer and its consequences such as short survival and poor prognosis requires the implementation of interventions that target factors identified through scientific investigations (research). Thus our review identified; first choice of traditional medicine, delay of > 3 months in seeking care, diagnosis or health system delay of > 2 months, rural residence, and chief complaint of painless breast lump as significantly associated factors to advanced-stage breast cancer diagnosis in Ethiopia. Patients who consider traditional medicine as their first choice before diagnostic confirmation are more likely to present with end-stage breast cancer as compared to their counterparts. The finding was in line with the study finding in Bangladesh [54], a systematic review of African countries [44], Malaysia [55], and Nigeria [56] in which patients who have experience of visiting traditional medicine before diagnostic confirmation are more frequently presented with advanced-stage breast cancer. This is because the time taken for traditional medicine visiting and trial may contribute to the advancement of the disease before diagnosis and come to the health system at the end stage. The problem might be more worrying in Ethiopia in which a large segment of the population utilizes traditional medicine and cancer patients are the most common visitors of traditional healers [57]. The finding suggests that intervention should be designed in awareness creation about breast cancer for traditional healers and improve their link with the health system to prevent advanced-stage presentation and poor prognosis.

In our review, patients who are delayed in seeking care are more frequently presented with advanced-stage breast cancer as compared to patients who seek care within 3 months of feeling symptoms. The finding was supported by evidence from a systematic review of African countries [44] and a global review [58] in which longer delays in seeking care were associated with more advanced stages of the diseases at diagnosis. Similarly in this review, we identified that patients who faced health system or diagnosis delay of greater than 2 months are prone to advanced stage at diagnosis as compared to patients who did not face diagnosis delay. The effect of this health system delay on advanced cancer diagnosis is also explained in a study in Mexico [52]. Both types of delay (delay from the patient in seeking care and delay in the health system or diagnosis delay) contribute to advanced-stage breast cancer presentation in the country. The finding implies efforts should be made to minimize the identified delays through mass awareness creation about early diagnosis of breast cancer and strengthen the early screening and prompt referral of cases in the lowest health care system of the country.

Moreover, in this review breast cancer patients from rural residences are twice as likely to be diagnosed at an advanced stage as compared to urban counterparts. The finding was supported by evidence from a systematic review of African countries [44], Nigeria [47], and two studies in Iran [49, 50] in which rural breast cancer patients are more frequently presented at a late stage of the disease that might greatly affect their survival. This might be due to the infrastructure in the rural environment does not permit them to present early or the environmental condition including the health care setup may expose them to both patient and health-system delays which leads them to late-stage presentation. Similarly, breast cancer patients who present with a chief complaint of painless breast lump were more than twice as likely to be diagnosed at an advanced stage as compared to patients who present with other chief complaints. A similar finding was reported in a systematic review of African countries [44] and a study in northern Pakistan [51]. This might be because a painless lump cannot be felt by patients and pushes them to seek treatment early. The finding suggests awareness creation on the signs and symptoms of breast cancer for the mass population is crucial and interventions that aimed at improving the early detection of breast cancer should more specifically target rural residents.

### Limitation of the study

In our review we have not made restrictions on publication date (until the last search date June 15, 2023) to included primary studies which might hide the current level of prevalence. We have included primary studies published from 2011 which is a 12 year period prevalence.

## Conclusion

More than two-thirds of the breast cancer cases in Ethiopia were diagnosed at an advanced stage, which indicates carefully planned interventions should be made to lower the proportion of patients diagnosed at the end stage of the disease and to enhance prognosis. Traditional medicine use before diagnostic confirmation, delay of > 3 months to seek care, diagnosis or health system delay of > 2 months, rural residence, and chief complaint of painless breast lump were positively associated with advanced–stage diagnosis. Therefore intervention efforts should focus on involving traditional healers, minimizing both patient and health system-related delays specifically targeting the rurally residing segment of the population so as to detect the disease early and improve survival.

### Electronic supplementary material

Below is the link to the electronic supplementary material.

Supplementary Material 1

Supplementary Material 2

Supplementary Material 3

## Data Availability

The result of this SRMA was extracted from the data gathered and analyzed based on the stated methods and materials. All the relevant data are within the paper.

## Abbreviations

PRISMA: Preferred Reporting Items for Systematic Reviews and Meta-Analyses
SNNPR: South Nation Nationality and People Region
SRMA: Systematic Reviews and Meta-Analyses
