# Supplementary material for: Advanced-stage breast cancer diagnosis and its determinants in Ethiopia: a systematic review and meta-analysis
Source: BMC Womens Health. 2024 May 11;24:284. doi: 10.1186/s12905-024-03133-9 (PMC11088059; doi:10.1186/s12905-024-03133-9)
Supplement: Supplementary file 2 — Supplementary Material 2 [file 12905_2024_3133_MOESM2_ESM.docx]

**Supplementary table 2 :** Newcastle-Ottawa Quality Assessment Scale for cross sectional studies used in the systematic review and meta-analysis 2023

|  | Selection | | | | Comparability | Outcome | | Total score |
| --- | --- | --- | --- | --- | --- | --- | --- | --- |
| Authors | Representativeness s (1) | Sample size (1) | Non respondents (1) | Ascertainment of the exposure (risk factor) (2) | The subjects in different outcome groups are comparable, based on the study design or analysis. confounding factors are controlled (1) | Assessment of the outcome (2) | Statistical test (1) |  |
| Gebremariam, et al. | 1 | 1 | 1 | 2 | 1 | 2 | 1 | 9 |
| Tesfaw, et al. | 1 | 1 | 1 | 2 | 1 | 2 | 1 | 9 |
| Tesfaw, et al. | 1 | 1 | 1 | 2 | 1 | 2 | 1 | 9 |
| Yoseph, et al. | 1 | 1 | 1 | 2 | 1 | 1 | 1 | 8 |
| Abebe, et al. | 1 | 0 | 1 | 2 | 1 | 2 | 0 | 7 |
| Areri, et al. | 1 | 1 | 1 | 2 | 1 | 2 | 1 | 9 |
| Assefa S. | 1 | 0 | 1 | 1 | 1 | 2 | 1 | 7 |
| Ayele, et al. | 1 | 1 | 1 | 1 | 1 | 2 | 1 | 8 |
| Belachew, et al. | 1 | 1 | 1 | 2 | 1 | 1 | 1 | 8 |
| Dagne, et al. | 1 | 1 | 1 | 2 | 1 | 1 | 1 | 8 |
| Gebretsadik,et al. | 1 | 1 | 1 | 2 | 1 | 1 | 1 | 8 |
| Gemta, et al. | 1 | 1 | 1 | 1 | 1 | 2 | 1 | 8 |
| Hassen, et al. | 1 | 1 | 1 | 1 | 1 | 2 | 1 | 8 |
| Legese, et al. | 1 | 1 | 1 | 2 | 1 | 1 | 1 | 8 |
| Muhammed,et al. | 1 | 1 | 1 | 1 | 1 | 2 | 1 | 8 |
| Shita, et al. | 1 | 1 | 1 | 2 | 1 | 1 | 1 | 8 |
| Solomon, et al | 1 | 0 | 1 | 2 | 1 | 1 | 1 | 7 |
| Tesfaw, et al. | 1 | 1 | 1 | 1 | 1 | 2 | 1 | 8 |
| Teshome, et al. | 1 | 1 | 1 | 2 | 1 | 1 | 1 | 8 |
| YOSEPH R. | 1 | 0 | 1 | 1 | 1 | 2 | 1 | 7 |
