# Supplementary material for: Advanced-stage breast cancer diagnosis and its determinants in Ethiopia: a systematic review and meta-analysis
Source: BMC Womens Health. 2024 May 11;24:284. doi: 10.1186/s12905-024-03133-9 (PMC11088059; doi:10.1186/s12905-024-03133-9)
Supplement: Supplementary file 3 — Supplementary Material 3 [file 12905_2024_3133_MOESM3_ESM.docx]

# Search strategy

**PubMed**

(((((((((((((((((("advanced"[All Fields] OR "advance"[All Fields]) OR "late"[All Fields]) OR "lately"[All Fields]) OR " delayed "[ All Fields]) OR "progressed"[All Fields]) OR "progressive"[All Fields]) OR " end-stage"[All Fields]) OR "last-stage"[All Fields]) AND "diagnosis"[All Fields]) OR "detection"[All Fields]) OR "identification"[All Fields]) OR "presentation"[All Fields]) OR "appearance"[All Fields]) OR "arrival"[All Fields]) OR " coming "[All Fields]) AND "breast"[All Fields]) AND (("cancer"[MeSH Terms] OR ""[All Fields]) OR "tumor"[All Fields])) OR "malignancy"[MeSH Terms]) OR [All Fields])) OR (("risk factors"[MeSH Terms] OR ("risk"[All Fields] AND "factors"[All Fields])) OR "risk factors"[All Fields])) OR " determinants "[ All Fields]) OR "causes"[All Fields]) OR "predictors"[All Fields]) OR "reasons” AND "Ethiopia"[All Fields])))

**Embase**

(“advanced stage diagnosis” OR “late stage diagnosis” OR “delayed presentation” OR “delayed diagnosis” AND “breast cancer” OR “breast tumor” OR “breast malignancy” AND Ethiopia AND [english]/lim NOT ([review]/lim OR [conference abstract]/lim OR [conference paper]/lim OR [letter]/lim OR [note]/lim OR [editorial]/lim OR [short survey]/lim OR [erratum]/lim OR 'case report'/de) NOT [animals]/lim

**Google scholar**

All in title: Advanced-stage breast cancer diagnosis AND associated factors in Ethiopia. All in title: "late stage breast cancer diagnosis " AND Ethiopia. All in title: "end-stage breast cancer diagnosis” AND Ethiopia. All in title: "determinants of " AND “Advanced-stage breast cancer diagnosis “ AND Ethiopia. All in title: “factors associated" AND " Advanced-stage breast cancer diagnosis " AND Ethiopia

**African Journals online**

“Advanced-stage breast cancer diagnosis and its determinants in Ethiopia”

“late-stage breast cancer diagnosis and its determinants in Ethiopia”
